# The Rare Adverse Effect of Cefepime-Induced Neutropenia

**DOI:** 10.7759/cureus.38274

**Published:** 2023-04-29

**Authors:** Omar Jumaah, Mohammad Abu-Abaa, Karen Huang, Saba Hasan

**Affiliations:** 1 Internal Medicine Residency Program, Capital Health Regional Medical Center, Trenton, USA

**Keywords:** cephalosporin bone suppression, cbc monitoring, neutropenic fever, cefepime-induced neutropenia, neutropenia

## Abstract

Cefepime is widely considered a safe and effective antibiotic, but it can have rare and serious side effects. We present a case of a 33-year-old female patient who developed severe and potentially life-threatening neutropenia after being on cefepime for 25 days. Despite extensive investigations, no other causes of neutropenia could be identified. Discontinuing the medication and administering a single dose of filgrastim produced a rapid and dramatic response. This case highlights the rare but serious risk of cefepime-induced neutropenia and underscores the need for clinicians to remain vigilant for this potential adverse effect. It is important to note that discontinuing the medication can rapidly reverse the effects, making timely recognition and intervention crucial for patient outcomes.

## Introduction

Cefepime has been recommended for the management of those with febrile neutropenia [1], mainly because of its broad-spectrum activity but also this is reflecting the low incidence of cefepime-induced neutropenia (CIN) and the benign nature of the medication [1]. It has the advantage of covering organisms such as *Pseudomonas *with relatively lower minimum inhibitory concentration (MIC) values as compared to other broad-spectrum beta-lactam antibiotics [1].

## Case presentation

A 33-year-old female patient presented to the emergency department (ED) with subjective fever at home, without any other complaints. Her past medical history is notable for a recent L5-S1 transforaminal interbody fusion three months prior to her current presentation. The bone graft was later removed due to a *Pseudomonas *wound infection and osteomyelitis one month prior to her current presentation, and she was placed on daily long-term cefepime (2 grams twice daily) through a peripherally inserted central catheter (PICC). Her past medical history was otherwise unremarkable. In the ED, her vital signs were recorded as follows: temperature 37.1 degrees Celsius, blood pressure 110/70 mmHg, heart rate 106 beats per minute with sinus rhythm, respiratory rate 18 cycles per minute, and SpO2 99% on room air. On physical examination, the surgical site was non-tender and non-erythematous, the PICC line site was non-tender and non-erythematous, the chest was clear on auscultation, and the abdomen was soft without tenderness. Otherwise, the examination was unremarkable. Basic labs showed leukopenia of 1.48 x 10^3/mcl (reference range 4-10x^3/mcl) and neutropenia with an absolute neutrophil count (ANC) of 0.04x10^^^3/mcl (reference range 1.6-6.1x10^^^3/mcl). Otherwise, her labs were unremarkable. The PICC line was removed, and cefepime was discontinued after 25 days of antibiotic therapy. Meropenem was initiated instead. Blood cultures and chest X-rays were negative. Screening duplex ultrasound for thrombosis at the site of the PICC line was negative, and the patient was hospitalized.

Within 24 hours of admission, the progression of neutropenia to an ANC of 0 was evident. A single dose of filgrastim was administered, which resulted in rapid improvement in her ANC to 3.90x10^3/mcl within the next 24 hours. No further doses of filgrastim were given, considering the improvement observed (see Table 1). Meropenem continued with a stable trend of ANC, allowing for safe discharge.

**Table 1:** Blood work results WBC = white blood cell, ANC = absolute neutrophil count, Hb = hemoglobin, Plt = platelet, BUN = blood urea nitrogen, Cr = creatinine, AST = aspartate aminotransferase, ALT = alanine transaminase, T. Bilirubin = total bilirubin

Variables	One month before admission	At admission	First day of admission	Second day of admission	Two weeks follow up
WBC	12.04	1.48	2.20	7.50	9.86
ANC	8.25	0.04	0.00	3.90	5.62
Hb	11.8	11.7	10.9	11.3	11.8
Plt	504	243	203	246	316
BUN	12	13	9	8	14
Cr	0.74	0.73	0.59	0.61	0.53
AST	27	43	42	40	28
ALT	22	24	24	23	23
T. Bilirubin	0.3	0.4	0.3	0.5	0.4

## Discussion

Neutropenia is a well-documented side effect of beta-lactam antibiotics, with an incidence of 10% within two weeks of initiation [2]. CIN has a prevalence of less than 1% according to post-marketing surveillance [3].

The mechanism of CIN remains largely unclear, although possible mechanisms have been suggested, including direct toxic effects on the bone marrow and immune-mediated mechanisms [4,5]. Dose-dependent inhibition of granulopoiesis was observed in studies of beta-lactam-induced neutropenia and supported by bone marrow findings [4,5]. Risk factors for CIN remain poorly understood but likely include a prolonged course of intravenous (IV) administration of more than two weeks [6]. Another reported risk factor is rapid IV push administration [7]. However, this was reported as a risk factor in long-term administration, as short-term administration of rapid IV push cefepime has been noted as a safe strategy [8].

The Naranjo scale, or adverse drug reaction (ADR) scale, was developed to standardize the assessment of causality in all potential ADRs [9]. Applying the Naranjo score to our case, the case receives one point for prior similar reports of CIN, two points for the temporal relationship between the initiation of the medication and the onset of neutropenia, one point for the resolution of neutropenia after medication discontinuation, and two points for the lack of other identifiable possible etiologies, totalling six points indicating the probability of CIN.

The cornerstone of the management of neutropenia secondary to beta-lactam antibiotics, including cefepime, is the discontinuation of the inciting medication [2]. In most of the reported cases of CIN, mere discontinuation of the medication allowed for the spontaneous recovery of ANC [6]. The prognosis is usually excellent. No clear information is available regarding the risk of recurrence; however, a literature analysis has found that the history of CIN is not a contraindication for future use of the medication as long as close laboratory monitoring is available [2].

## Conclusions

Cefepime is a valuable tool in the treatment of bacterial infections due to its broad-spectrum activity and relative safety profile. However, the potential for CIN, although rare, should not be overlooked. Clinicians must remain vigilant and consider this as a potential diagnosis in patients receiving cefepime who develop unexplained neutropenia. Early recognition and prompt discontinuation of the medication can lead to rapid recovery and prevent potentially life-threatening complications. Overall, careful consideration of the benefits and risks of cefepime use is essential in optimizing patient outcomes.
